# A simple-to-use score system for predicting HBsAg clearance to peginterferon alfa-2b in nucleoside analogs-experienced chronic hepatitis B patients

**DOI:** 10.3389/fmed.2023.1243202

**Published:** 2023-11-27

**Authors:** Kaimin Song, Dawu Zeng, Yijuan Zheng, Huatang Zhang, Zhangyan Weng, Yongjun Zhou, Zhijun Su, Xueping Yu

**Affiliations:** ^1^Department of Infection Disease, Fujian Medical University Affiliated First Quanzhou Hospital, Quanzhou, Fujian, China; ^2^Department of Liver Center, The First Hospital Affiliated to Fujian Medical University, Fuzhou, China; ^3^College of Life Sciences and Chemistry, Institute of Bioengineering and Biotechnology, Minnan Science and Technology University, Quanzhou, China

**Keywords:** hepatitis B, chronic, hepatitis B surface antigens, peginterferon alfa-2b, regression analysis, aspartate aminotransferases

## Abstract

**Objective:**

Patients with chronic hepatitis B (CHB) often fail to achieve clearance of the hepatitis B surface antigen (HBsAg) with peginterferon treatment. Our study aimed to develop a simple-to-use scoring system to predict the likelihood of HBsAg clearance following treatment with peginterferon alfa-2b(PEG-IFN-α2b) in patients with CHB.

**Methods:**

A total of 231 patients were enrolled and divided into HBsAg clearance (*n* = 37) and non-HBsAg clearance (*n* = 194) groups. Multifactor logistic models were constructed using univariate and multiple logistic regression analyses. The area under the receiver operating characteristic curve (AUC), calibration curve, and decision curve analysis were used to evaluate the discrimination, calibration, and clinical applicability of the predictive scoring system.

**Results:**

Four clinical variables (age, baseline HBsAg level, HBsAg level decline at week 12, and alanine aminotransferase ratio at week 12) were independently associated with HBsAg clearance after PEG-IFN-α2b treatment and, therefore, were used to develop a predictive scoring system ranging from 0 to 13. The optimal cut-off value was >4, with a sensitivity of 86.49%, specificity of 72.16%, positive predictive value of 37.2%, negative predictive value of 96.6%, and an AUC of 0.872. This model exhibited good discrimination, calibration, and clinical applicability. Among patients with scores <4, 4, or > 4 HBsAg clearance was achieved in 0.85, 14.29, and 37.21% of the patients, respectively.

**Conclusion:**

The scoring system could effectively predict the predominance of HBsAg clearance after PEG-IFN-α2b treatment in the early stage. This may be helpful when making clinical decisions for the treatment of patients with CHB.

## Introduction

Despite the widespread availability of vaccines and the use of highly effective antiviral drugs, chronic hepatitis B virus (HBV) infection remains a major global public health problem, affecting approximately 257 million people (1–3) and causing 650,000 deaths worldwide each year from cirrhosis, liver failure, and hepatocellular carcinoma due to HBV (4). For patients with chronic hepatitis B (CHB), aggressive and effective treatment is essential to prevent disease progression and improve the long-term prognosis. HBV enters the liver cells and forms covalently closed circular deoxyribonucleic acid (cccDNA) and integrated HBV-DNA, which play important roles in chronic viral infections and disease persistence (5). Given that complete clearance of cccDNA and integrated HBV-DNA is almost impossible to achieve, experts recommend functional or clinical cures as the ideal treatment goal for CHB, which is defined as hepatitis B surface antigen (HBsAg) clearance with or without the presence of anti-HBs and persistent undetectable HBV-DNA (6). HBsAg clearance is a marker of significant suppression of HBV replication and cccDNA transcription. Studies have reported that HBsAg clearance reduces the risk of liver cancer by 5-fold in patients with CHB compared with HBsAg-positive patients (7). This also makes HBV reactivation very unlikely (8). Therefore, HBsAg clearance has become a priority in the management of patients with CHB.

The rate at which patients with CHB spontaneously become negative for HBsAg is extremely low, at approximately 1% per year (9). The rate of HBsAg clearance after treatment with potent nucleotide analogs has been reported to be <3% (10). By contrast, interferons, as immunomodulatory agents, have shown better results for HBsAg clearance. Research has shown that in nucleoside analogs (NAs)-treated patients, HBsAg clearance can reach 14.4% after 48 weeks of sequential switching to peginterferon treatment, and up to 20.7% after 96 weeks (11). However, peginterferons have some limitations, including high cost and side effects. Additionally, a large proportion of patients continue to remain HBsAg-positive even after experiencing the side effects of peginterferon (12). Therefore, it has become important to identify specifically which patients, among the many with CHB, are most or least likely to achieve HBsAg clearance after peginterferon treatment. Studies (7, 11, 12) have explored predictors of HBsAg clearance; however, these have mostly been single indicators or ones that were difficult to obtain routinely, and the complex interactions between multiple influencing factors were largely ignored. In this study, we selected patients with CHB broadly, and extracted common clinical variables among them, based on electronic medical records, to explore the factors associated with HBsAg clearance following treatment with peginterferon alfa-2b(PEG-IFN-α2b). We then combined multiple influencing factors to develop a predictive model, and translated it into a scoring system as a simple-to-use tool to assist in clinical decision-making.

## Methods

### Study population and design

A total of 990 patients with CHB who were treated with PEG-IFN-α2b (180 g/week) at Fujian Medical University Affiliated First Quanzhou Hospital (Quanzhou, China) between January 2016 and December 2021 were retrospectively screened. All patients received NAs treatment prior to PEG-IFN-α2b. According to the Guidelines for the prevention and treatment of chronic hepatitis B (version 2022) (13) and optimizing-seroconversion sequential treatment (OSST) trial from China (14, 15), the patients received “add-on” or “switch-to” treatment strategy. For the PEG-IFN-α2b “add-on” therapy, all patients received entecavir (ETV), tenofovir disoproxil fumarate (TDF) or tenofovir alafenamide fumarate(TAF) combined with PEG-IFN-α2b. The “switch-to” group meant patients received PEG-IFN-α2b therapy singly. The inclusion criteria were as follows: (1) serum HBsAg present for 6 months or more; (2) antiviral treatment with NA for at least 12 weeks previously; (3) age between 18 and 65 years; and (4) PEG-IFN-α2b treatment for at least 48 weeks and up to 72 weeks, with complete follow-up data. The exclusion criteria were as follows: (1) coinfection with hepatitis C virus (HCV), hepatitis D virus (HDV), hepatitis E virus (HEV), or human immunodeficiency virus (HIV); (2) discontinuation or change in treatment regimen; (3) concomitant history of cirrhosis, hyperthyroidism, thyroiditis, autoimmune hepatitis, pregnancy, or any type of neoplasm; and (4) incomplete relevant test data or information. Ultimately, 231 patients with CHB were included in this study (Supplementary Figure S1).

The patients were divided into groups with and without HBsAg clearance according to the treatment outcome. HBsAg clearance was defined as the disappearance of HBsAg (<0.05 IU/mL) in previously HBsAg-positive patients; HBV-DNA negativity was defined as HBV-DNA below the lower limit of detection (<20 IU/mL); and the alanine aminotransferase (ALT) ratio was defined as a multiple of the upper limit of the normal (50 IU/L). This single-center retrospective study was approved by the Research Ethics Committee of Fujian Medical University Affiliated First Quanzhou Hospital, and followed the principles of the Declaration of Helsinki.

### Data collection

General information about each patient included name, sex, age, whether they were treatment-naïve, treatment strategy, route of HBV Infection, and the duration of PEG-IFN-α2b treatment. HBsAg, anti-HBs, HBeAg, anti-HBe, and anti-HBc levels were quantified by chemiluminescent particulate immunoassays using the Architect i2000 SR platform and Abbott Architect reagents (Abbott Laboratories, Chicago, IL, United States). The COBAS AmpliPrep/COBAS TaqMan system (Roche Diagnostics, Mannheim, Germany) was used to detect serum HBV-DNA by quantitative polymerase chain reaction, with a lower limit of detection of 20 IU/mL. During PEG-IFN-α2b treatment, the levels of HBsAg, anti-HBs, HBeAg, anti-HBe, anti-HBcAb, HBV-DNA, ALT, aspartate aminotransferase, albumin, globulin, total bilirubin, alkaline phosphatase, glutamyl transpeptidase, and hemoglobin, as well as white blood cell, red blood cell, and platelet counts, were recorded at each 12-week follow-up visit from the start of treatment until the endpoint.

### Statistical analyses

All statistical analyses were performed using SPSS 27.0 (IBM Corporation, Armonk, NY, United States) and R software version 4.2.2(R core development team, Vienna, Austria). Categorical variables are expressed as frequencies or percentages (%), continuous variables conforming to a normal distribution are expressed as mean ± standard deviation, and continuous variables not conforming to a normal distribution are expressed as median (M) and interquartile range (IQR). Depending on the distribution of the data, the significance of differences in continuous variables was checked using a non-parametric test (Mann–Whitney U test) or a two-independent samples Student’s *t*-test. Categorical variables were compared using the chi-squared test or Fisher’s exact probability method. Results were considered statistically significant at *p* < 0.05.

The development of the predictive scoring system involved the following steps: First, patient demographics and baseline and clinical characteristics at week 12 of treatment were included, and the relationship between these variables and treatment endpoint HBsAg clearance was explored using univariate logistic regression, with variables meeting *p* < 0.1 selected for the next step of the analysis. For this process, the receiver operating characteristic (ROC) curve was plotted to determine the optimal cut-off values for baseline HBsAg and HBsAg levels at week 12, which were converted to categorical variables. Independent factors associated with HBsAg clearance were identified using stepwise multiple logistic regression analysis. Subsequently, variables with *p* < 0.05 were selected to construct multiple logistic regression models. Finally, a predictive score system was generated by assigning scoring points according to the magnitude of the regression coefficients in the logistic regression equation according to the method described by Sullivan and colleagues in their Framingham risk scoring study (16). The constructed predictive scoring system was internally validated using the bootstrap method, and the optimal cut-off value, sensitivity, specificity, positive predictive value, and negative predictive value of the score system were calculated. In addition, the area under the receiver operating characteristic curve (AUC) was used to assess the discrimination of the score system. The Hosmer–Lemeshow test and calibration curve were used to determine the calibration, and decision curve analysis (DCA) was used to evaluate clinical applicability. In addition, in order to better evaluate the score system, subgroup analysis was performed according to the route of HBV infection.

## Results

### Demographic characteristics

A total of 231 patients with CHB were included in this study. Their median age was 37.00 (IQR, 31.00–44.00), the HBsAg titer was 3.07 lg IU/mL at baseline, the HBV-DNA load was 2.49 lg IU/mL at baseline, the ALT was 58 U/L at baseline. Among them, 76.19% were male, 45.89% were HBeAg positive, 40.26% were HBV-DNA negative prior to treatment, 45.89% were infected through vertical transmission, and the proportion of patients who received “add-on” treatment programs was 48.05%. Moreover, prior to starting PEG-IFN-α2b, 28.6% patients had received ETV, 48.5% had received TDF and 22.9% had received TAF. At the end of PEG-IFN-α2b treatment, 37 patients achieved HBsAg clearance, indicating an overall clearance rate of 16.02%. In terms of demographic characteristics, the proportion of HBeAg-positive patients was significantly lower in the group with HBsAg clearance than in the group without, and HBsAg was significantly lower in the HBsAg clearance group. No significant differences were observed in the remaining characteristics (Table 1).

**Table 1:** Demographic and clinical characteristics at baseline.

Characteristic	Entire cohort (*n* = 231)	With HBsAg clearance (*n* = 37)	Without HBsAg clearance (*n* = 194)	Z/t/χ^2^	*P*
Age, years (median, IQR)	37.00 (31.00–44.00)	34.00 (28.00–42.50)	37.00 (31.00–44.25)	1.816	0.069
BMI (kg/m^2^)	24.35 ± 3.35	24.43 ± 3.16	24.34 ± 3.39	−0.145	0.885
Male sex (%)	76.19%	75.7%	76.3%	0.006	0.936
HBeAg positive (%)	45.89%	29.7%	49.0%	4.632	**0.031**
Treatment strategy, add-on (%)	48.05%	59.5%	45.9%	2.297	0.130
HBV-DNA negative (%)	40.26%	51.4%	38.1%	2.254	0.133
Route of HBV Infection, vertical transmission (%)	45.89%	43.2%	46.4%	0.124	0.725
HBsAg (lg, IU/mL)	3.07 (2.52–3.58)	2.33 (1.77–2.95)	3.15 (2.73–3.70)	−4.735	**<0.001**
HBV-DNA (lg, IU/mL)	2.49 (1.00–4.92)	1.28 (1.26–3.93)	2.68 (1.00–5.29)	−0.268	0.789
ALT (U/L)	58.00 (54.00–96.00)	56.00 (54.50–89.00)	58.00 (54.00–100.75)	−0.672	0.501
AST (U/L)	32.00 (24.00–55.00)	28.00 (21.00–51.00)	33.00 (25.00–55.00)	−1.564	0.118
ALB (g/L)	44.90 (42.80–46.40)	45.20 (43.25–47.15)	44.80 (42.48–46.33)	−0.933	0.351
PLT (10^9/L)	210.00 (167.00–249.00)	228.00 (192.00–256.50)	206.50 (161.75–247.25)	−1.612	0.107
LSM (kPa)	8.10 (7.60–10.00)	8.60 (7.70–10.05)	8.10 (7.55–10.00)	−1.220	0.222
Duration of NAs treatment(weeks)	48.00 (48.00–72.00)	48.00 (48.00–72.00)	52.00 (48.00–72.00)	−0.914	0.361
NAs classification					
ETV (%) TDF (%) TAF (%)	28.6% 48.5% 22.9%	16.2% 56.8% 27.0%	30.9% 46.9% 22.2%	3.296	0.192
HBsAg clearance (%)	16.02%	–	–	–	–

HBeAg, hepatitis B e antigen; HBsAg, hepatitis surface antigen; HBV-DNA, hepatitis B virus-deoxyribonucleic acid; IQR, interquartile range; NAs, nucleoside analogs; ETV, entecavir; TDF, tenofovir disoproxil fumarate; TAF, tenofovir alafenamide fumarate; BMI, body mass index; ALT, alanine aminotransferase; AST, aspartate aminotransferase; ALB, albumin; PLT, platelet; LSM, Liver stiffness measurement. Bold values represent *p*-value that meet statistical test levels.

### Selection of predictors for HBsAg clearance

ROC curves were used to determine the optimal cut-off values for baseline HBsAg and HBsAg levels at week 12. These were determined to be: baseline HBsAg ≤605 IU/mL and HBsAg at week 12 ≤ 77 IU/mL. Patient characteristics at baseline and week 12 were then subjected to univariate logistic regression analysis with *p* < 0.1 as the criterion to select eligible characteristics for next multiple logistic regression analysis. Independent factors associated with HBsAg clearance were thus identified as follows: age, baseline HBsAg level, HBsAg level decline at week 12, and ALT ratio at week 12 (Table 2).

**Table 2:** Univariate and multiple logistic regression of factors associated with HBsAg clearance.

Characteristic	Univariate logistic regression			Multiple logistic regression		
	Regression coefficient	Odds ratio	*P*	Regression coefficient	Odds ratio	*P*
Age (years)	−0.039	0.961	0.056	−0.072	0.930	**0.021**
Duration of PEG-IFN-α2b treatment (weeks)	−0.033	0.967	0.081	0.015	1.015	0.533
HBeAg status (positive vs. negative)	0.819	2.268	0.034	0.232	1.261	0.719
Baseline HBsAg (>605 IU/mL vs. ≤605 IU/mL)	2.113	8.277	<0.001	2.496	12.130	**<0.001**
Baseline TBil (μmol/L)	−0.058	0.944	0.085	−0.042	0.959	0.344
HBsAg at week 12(>77 IU/mL vs. ≤77 IU/mL)	2.472	11.850	<0.001	−0.457	0.633	0.524
HBsAg decline at week 12(log_10_ IU/mL)	1.154	3.170	<0.001	1.095	2.990	**<0.001**
HBV-DNA status at week 12 (negative vs. positive)	0.849	2.337	0.033	−0.064	0.938	0.921
ALT ratio at week 12 (ULN)	0.460	1.584	0.003	0.402	1.495	**0.034**

ALT, alanine aminotransferase; HBeAg, hepatitis B e antigen; HBsAg, hepatitis surface antigen; HBV-DNA, hepatitis B virus-deoxyribonucleic acid; TBil, total bilirubin; ULN, upper limit of the normal. ALT ratio means ALT/ULN. Bold values represent *p*-value that meet statistical test levels.

### Development of a predictive score system

These four independent risk factors were included, and stepwise multiple logistic regression was used to construct a model with the following results: age [odds ratio (OR) =0.933, *p* = 0.017], baseline HBsAg level (OR = 10.296, *p* < 0.001), HBsAg level decline at week 12 (OR = 2.590, *p* < 0.001), and ALT ratio at week 12 (OR = 1.453, *p* = 0.041). Scoring points were assigned according to the magnitude of the regression coefficients (Supplementary Table S1). The final score ranged from 0 to13, with higher total scores indicating a greater likelihood of achieving HBsAg clearance in patients with CHB after the end of PEG-IFN-α2b treatment (Table 3).

**Table 3:** Score system for HBsAg clearance of CHB patients treated with peginterferon alfa-2b.

Predictors	Categories	Points
Age(years)	≥50	0
	40–49	1
	30–39	2
	<30	3
Baseline HBsAg (IU/mL)	>605	0
	≤605	3
HBsAg decline at week 12(log_10_ IU/mL)	≤0.5	0
	0.5–1	1
	>1	4
ALT ratio at week 12 (ULN)	<2	0
	2–5	1
	≥5	3

ALT, alanine aminotransferase; HBsAg, hepatitis B surface antigen; ULN, upper limit of the normal. ALT ratio means ALT/ULN.

### Performance evaluation of the predictive score system

**Figure 1:**
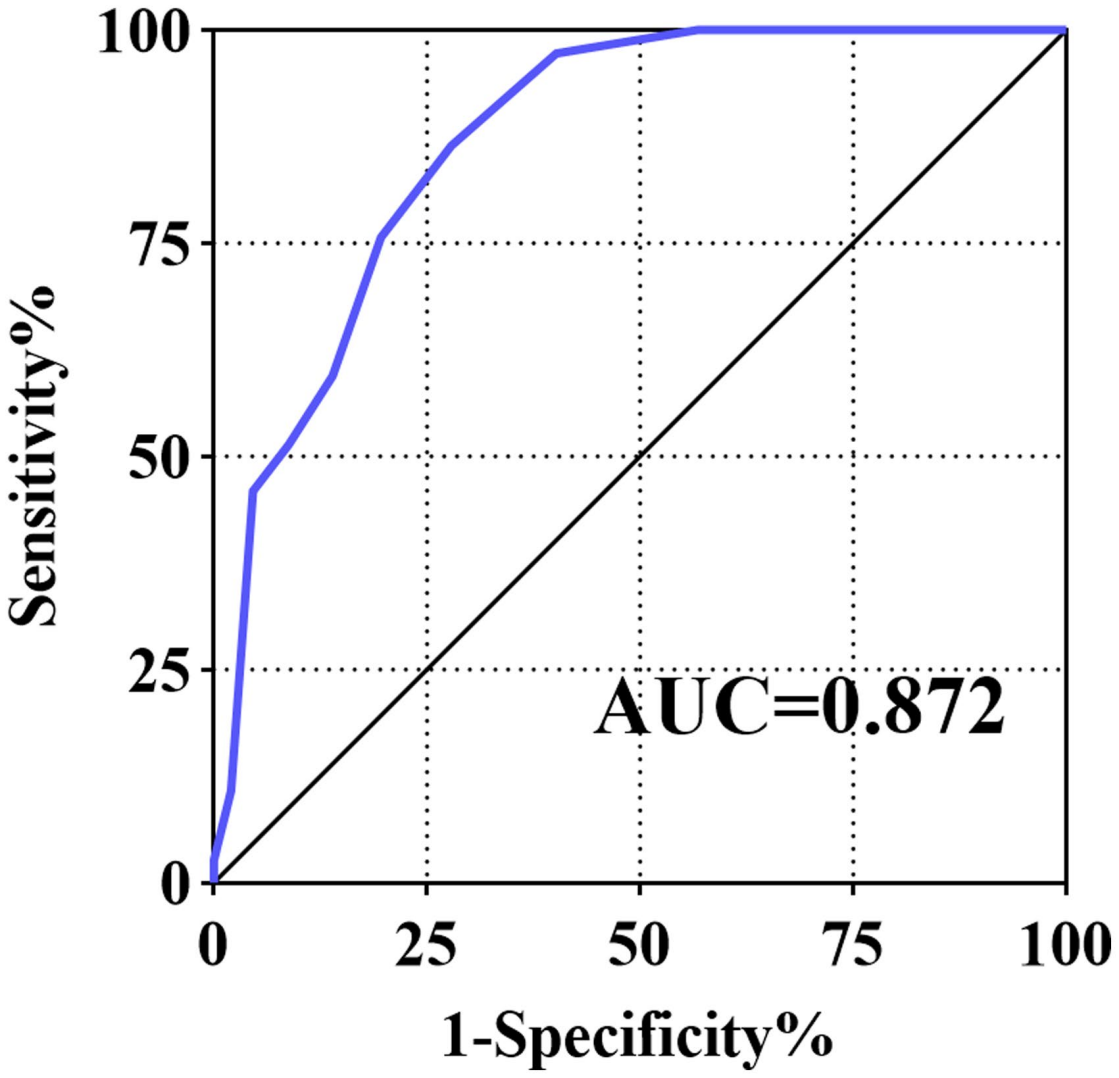
Receiver operating characteristic curve for the score system.

The optimal cut-off value for the scoring system was >4, with a sensitivity of 86.49%, specificity of 72.16%, positive predictive value of 37.2%, and negative predictive value of 96.6%. The AUC was 0.872 (95% confidence interval [*CI*]:0.822–0.912) in the cohort and 0.896 (95% CI:0.895–0.898) for internal validation using the bootstrap method, suggesting that the scoring system had good discrimination performance (Figure 1). In terms of our Hosmer–Lemeshow test (χ^2^ = 3.640), *p* = 0.888, combined with the calibration curve plots, suggested that the model had an adequate goodness of fit (Supplementary Figure S2). The DCA curves indicated that the model may have a broad clinical applicability (Supplementary Figure S3).

### Subgroup analysis on route of HBV infection of the score system

**Figure 2:**
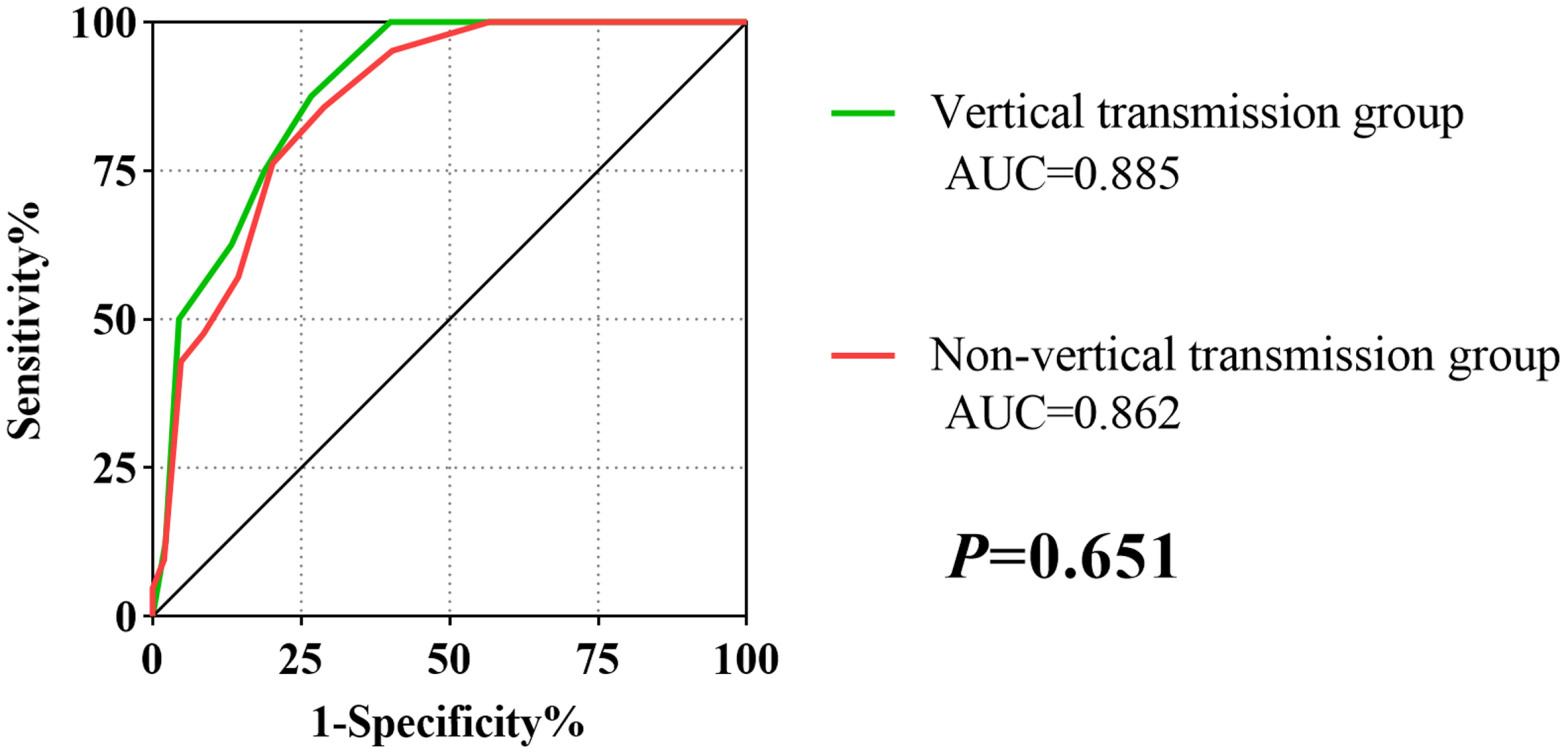
The ROC curves of the score model in vertical transmission and Non-vertical transmission subgroup.

As shown in Table 4, the score system had an AUC of 0.885 (95% *CI*: 0.808–0.939), a sensitivity of 87.50%, a specificity of 73.33%, a PPV of 36.80%, and a NPV of 97.10% in the vertical transmission subgroup. In the non-vertical transmission subgroup, the AUC was 0.862 (95% *CI*:0.789–0.917), with a sensitivity of 85.71%, specificity of 71.15%, PPV of 37.50% and NPV of 96.10%. According to the results of DeLong’s test, the scoring system had good stability and showed consistent performance in the vertical transmission subgroup and the non-vertical transmission subgroup (*p* = 0.651) (Figure 2).

**Table 4:** Subgroup analysis on route of HBV Infection of the score system.

Subgroups	AUC	*P*	Cut-off value	SEN	SPE	PPV	NPV
Vertical transmission	0.885 (0.808–0.939)	<0.001	>4	87.50%	73.33%	36.80%	97.10%
Non-vertical transmission	0.862 (0.789–0.917)	<0.001	>4	85.71%	71.15%	37.50%	96.10%

SEN, sensitivity; SPE, specificity; PPV, positive predictive value; NPV, negative predictive value; AUC, area under the ROC curve.

### Application of the predictive score system

**Figure 3:**
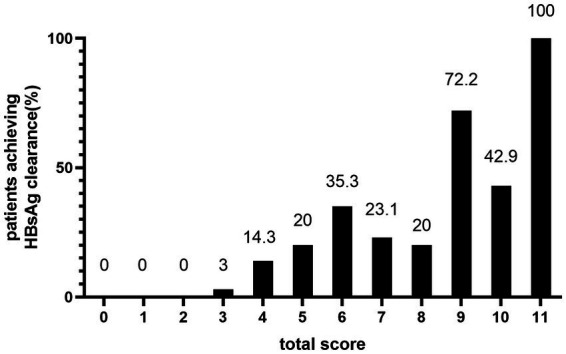
HBsAg clearance rate at EOT of peginterferon alfa-2b according to the predictive score system.

In the total patient cohort, the distribution of total scores was: 0 (*n* = 10, 4.33%); 1 (*n* = 32, 13.85%); 2 (*n* = 42, 18.18%); 3 (*n* = 33, 14.29%); 4 (*n* = 28, 12.12%); 5 (*n* = 20, 8.66%); 6 (*n* = 17, 7.36%); 7 (*n* = 13 5.63%); 8 (*n* = 10, 4.33%); 9 (*n* = 18, 7.79%); 10 (*n* = 7, 3.03%); and 11 (*n* = 1, 0.43%). None of the patients had scores of 12 or 13. As shown in Figure 3, there was an overall increasing trend in the proportion of patients who achieved HBsAg clearance at the end of PEG-IFN-α2b treatment as the total score increased. The proportion of patients who achieved HBsAg clearance was 0%, with total scores of 0, 1, and 2. In contrast, 100% achieved HBsAg clearance with a score of 11. However, because only one person had a total score of 11, it was not possible to accurately estimate the treatment response.

**Figure 4:**
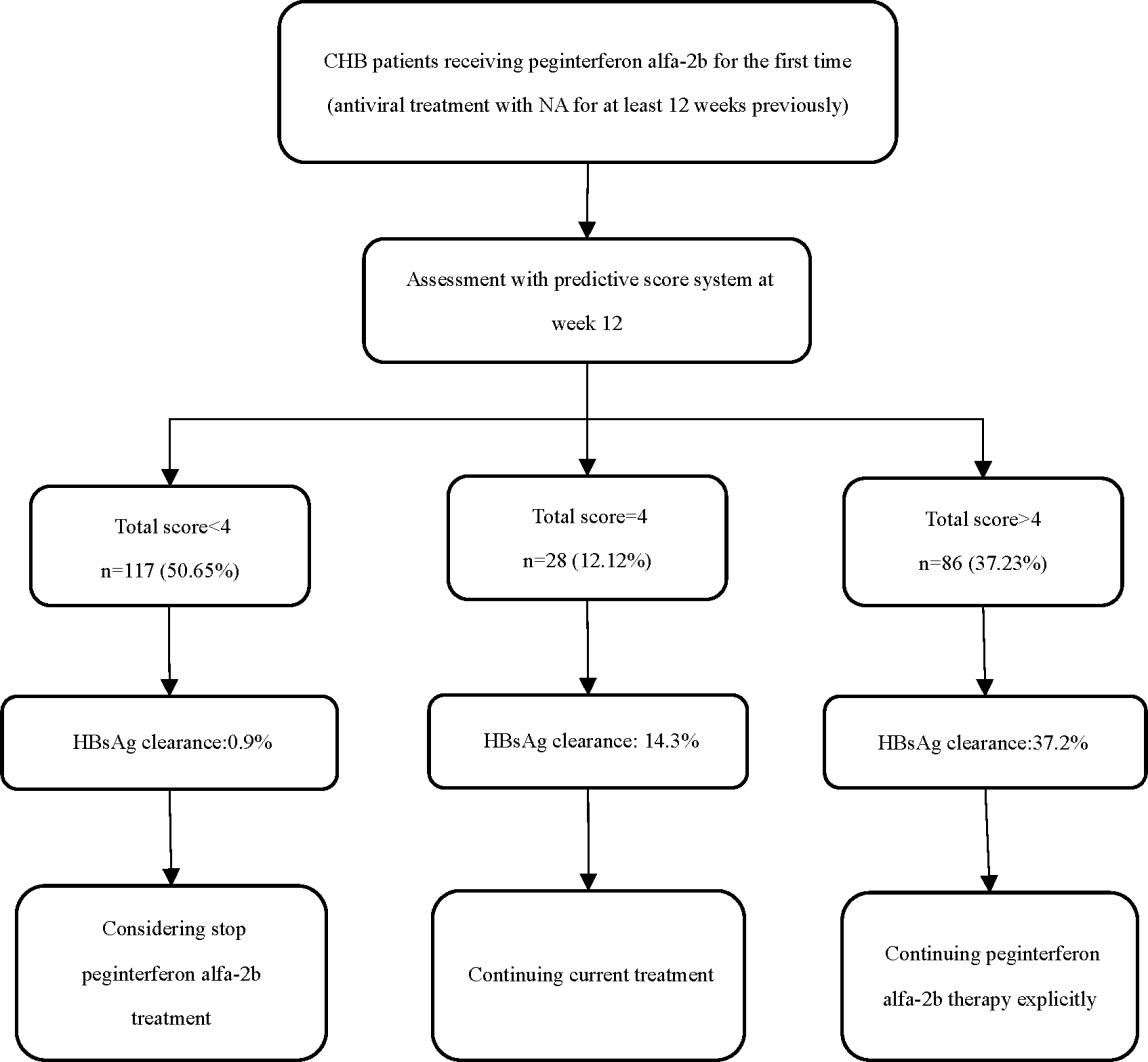
Suggested strategies of score system in the management of CHB patients treated with peginterferon alfa-2b.

Therefore, in combination with the optimal cut-off values from the score model, we divided the patients into three groups based on their total scores: <4, 4, or > 4 (Figure 4). After 48–72 weeks of PEG-IFN-α2b treatment, the proportion of patients with CHB who had scores of 0–3 and achieved HBsAg clearance was 0.85% (1/117), which was significantly lower than the overall cohort proportion of 16.02% (*p* < 0.001). The proportion of patients with a score of 4 was 14.29% (4/28), similar to the overall rate (*p* = 0.813). Among patients with a total score > 4, the HBsAg clearance rate (32/86) was significantly higher than the overall rate (37.21% vs. 16.02%, *p* < 0.001).

## Discussion

HBsAg clearance can be achieved by optimizing the treatment regimens used for patients with CHB. Using clinical demographic characteristics and routine laboratory tests, we developed a simple and useful scoring system for the early prediction of the likelihood of achieving HBsAg clearance at the end of PEG-IFN-α2b treatment in patients with CHB. This scoring system is based on four clinical variables (age, baseline HBsAg level, HBsAg level decline at week 12, and ALT ratio at week 12) and does not require expensive or difficult-to-perform testing techniques. Using this system, patients can be assessed individually and comprehensively approximately 12 weeks after starting PEG-IFN-α2b treatment to identify those who are most or least likely to achieve HBsAg clearance. This model is also easy to use in primary and community hospitals with limited access.

The HBsAg clearance rate for the overall population in our study was 16.02%, which was slightly higher than the HBsAg clearance rates of 14.4% (HBeAg-negative patients) and 8.5% (HBeAg-positive patients) reported in previous studies (11, 14). After univariate and multiple logistic regression analyses, we identified four factors that were independently associated with HBsAg clearance: age, baseline HBsAg level, HBsAg level decline at week 12, and the ALT ratio at week 12. The role of age in achieving a functional cure in patients with CHB has been reported previously. Yang et al. (17) showed that age was independently associated with HBsAg clearance after 48 weeks of interferon add-on therapy in patients with low baseline HBsAg levels. Further analysis revealed that HBsAg clearance after interferon treatment did not exceed 5% in patients older than 53.5 years, in contrast to patients younger than 30.5 years who had an HBsAg clearance of more than 95%. Similarly, we observed that age was an independent predictor of HBsAg clearance following PEG-IFN-α2b treatment. Older age was associated with lower scores on the scoring system, implying a lower likelihood of HBsAg clearance. The mechanisms underlying the relationship between the two are not yet clear. It has been demonstrated that age is a key factor affecting the function of HBV-specific CD4+ and CD8+ T cells, which are the main effector cells in controlling HBV infection, and that HBsAg-specific T cells account for 28.26% of total HBV-specific T cells in patients under 30 years of age, while this percentage drops to 7.14% in patients older than 30 years old (18, 19). Therefore, it is possible that the number and function of immune cells in the body decline with age, weakening the immunomodulatory function of interferons. Elevated ALT levels are considered a sign of an activated immune state and have been shown to be closely associated with HBsAg clearance achieved with interferon therapy (20). In the present study, we identified elevated ALT levels after 12 weeks of treatment as a significant predictor of HBsAg clearance, which is consistent with the approaches used in a number of previous studies (21–23).

Baseline HBsAg levels and the magnitude of early decline in HBsAg levels during antiviral therapy are valid markers for predicting HBsAg clearance, and can help identify different populations, in terms of responsiveness, during the course of interferon therapy. One multicenter randomized controlled trial reported that, among patients who switched from NAs to peginterferon alfa-2a treatment, baseline HBsAg levels of <1,500 IU/mL and HBsAg levels of <200 IU/mL at week 24 were associated with the highest rate of HBsAg loss at the end of 48 weeks of treatment (11). Wang et al. (12) observed that, in patients treated with NAs to achieve HBV-DNA conversion, the baseline HBsAg level and HBsAg level decline at week 12 were independent predictors of achievement of HBsAg clearance with sequential peginterferon combination therapy. They proposed that the optimal cut-off values for the two metrics were a baseline HBsAg level < 400 IU/mL and HBsAg level decline >1 log_10_ IU/mL at week 12. In patients with inactive chronic HBV carriers, Wu et al. (22) explored factors affecting HBsAg clearance with peginterferon alpha treatment, and found that patients with a baseline HBsAg level < 2.53 log_10_ IU/mL and a HBsAg level decline at week 12 > 0.4 log_10_ IU/mL were more likely to achieve HBsAg clearance at the endpoint of treatment. Similarly, our study showed that baseline HBsAg levels and early changes in treatment independently correlated with HBsAg clearance. However, the optimal cut-off values for baseline HBsAg levels in this study differed from those of previous studies. This may be due to the pre-treatment status of the study population, the treatment regimen, or the type of interferon used.

Using the Framingham model to build an integrated system, we developed a predictive scoring system based on a multiple logistic regression model that contained four variables. The optimal cut-off value of the model was >4, with high sensitivity (86.49%), specificity (72.16%), and negative predictive value (96.6%). The model also demonstrated good discrimination, calibration and clinical applicability, as well as good robustness in subgroup analysis. To further facilitate clinical practice, we divided the total score into three levels: <4, 4 or > 4. As the probability of achieving HBsAg clearance at the endpoint of treatment was quite low in the <4 group, it is recommended that these patients discontinue interferon therapy as early as possible and switch to other treatment regimens, since they are not a favored population for PEG-IFN-α2b treatment. In contrast, patients with score > 4 are strongly recommended to continue their current PEG-IFN-α2b regimen. Furthermore, the probability of achieving HBsAg clearance in the =4 population is similar to that of the overall population, so continuation of the current therapy is recommended for those patients. Since our scoring system makes a judgment quite early in the treatment course, the score 4 population can make further decisions based on treatment response during subsequent treatment periods (i.e., after 12 weeks of treatment), which can maximize the likelihood of avoiding exposure to adverse drug reactions and ensure the patient’s chances of a clinical cure. Past studies have reported several factors that influence the achievement of a functional cure using interferon. These factors include age, sex, baseline HBsAg, quantitative hepatitis B core antibody, and genotyping, but their value in predicting interferon efficacy is not clear when considering multiple factors acting together or interacting with one another. Unlike previous studies, our study included a broader spectrum of patient populations and increased clinical acceptability by stratifying the three continuous variables of age, HBsAg level decline at week 12, and ALT ratio at week 12, to better quantify the extent of their impact on treatment outcomes, as well as convert the continuous variable of baseline HBsAg level into a dichotomous one. The advantage of our proposed scoring system is that it integrates several routine clinical variables and early patient response to treatment for a wide range of patients with CHB who are treated using PEG-IFN-α2b, allowing physicians to predict individual endpoints more accurately in the early stages of treatment and take early action to ensure maximum effectiveness of antiviral therapy. Notably, our scoring system achieved a negative predictive value of over 96%, indicating that this model is highly effective in identifying people who are unlikely to achieve clinical cure with PEG-IFN-α2b. This can help patients minimize treatment exposure and potential adverse effects, while also saving healthcare resources to some extent.

However, this study also has several key limitations. First, the study was a single-center retrospective study, and only 231 patients out of over 900 were screened for inclusion in the final study; therefore, there may have been a selection bias, and the sample size may have been insufficient. Second, although the score system we developed was internally validated and showed reliable performance, the lack of external validation reduced the applicability of the model, so further validation in patients of different backgrounds is warranted. In addition, our study aimed to treat patients with PEG-IFN-α2b. Whether the model is applicable to other types of interferons therefore requires further validation. Finally, we did not include information on HBV genotyping or HBV-RNA, for example, in view of the simplicity and broad utility of the scoring system. In the future, we aim to further test some of these other viral markers, such as genotype.

In summary, we found that independent factors that affected the achievement of HBsAg clearance in patients with CHB who were treated with PEG-IFN-α2b included age, baseline HBsAg level, HBsAg level decline at week 12, and the ALT ratio at week 12. Our simple-to-use multifactor scoring system can be used early in treatment to identify patient populations in which PEG-IFN-α2b-based regimens are advantageous. This can help reduce unnecessary drug exposure and the occurrence of side effects, as well as facilitate the individualized management of CHB patients.

## Conclusion

We developed a scoring system that effectively predicted the predominance of HBsAg clearance following PEG-IFN-α2b treatment in the early stage. This system may be helpful when making clinical decisions for the treatment of patients with CHB.

## Data availability statement

The datasets presented in this article are not readily available because the data relates to the privacy of the patient. Requests to access the datasets should be directed to XY, xpyu15@fudan.edu.cn.

## Ethics statement

The studies involving humans were approved by the Research Ethics Committee of Fujian Medical University Affiliated First Quanzhou Hospital. The studies were conducted in accordance with the local legislation and institutional requirements. The participants provided their written informed consent to participate in this study.

## Author contributions

YoZ, ZS, and XY: study concept and design and critical revision of the manuscript for important intellectual content. KS, DZ, and YiZ: acquisition of data. KS, DZ, and YiZ: analysis and interpretation of data and statistical analysis. KS: drafting of the manuscript. HZ and ZW: administrative, technical, and material support. All authors checked the results, approved the final manuscript, contributed to the article and approved the submitted version.
