# Supplementary material for: A simple-to-use score system for predicting HBsAg clearance to peginterferon alfa-2b in nucleoside analogs-experienced chronic hepatitis B patients
Source: Front Med (Lausanne). 2023 Nov 27;10:1243202. doi: 10.3389/fmed.2023.1243202 (PMC10711601; doi:10.3389/fmed.2023.1243202)
Supplement: Supplementary file 1 [file Data_Sheet_1.docx]

**

**

**Figure S1** Flow diagram of participant recruitment. CHB, chronic hepatitis B; HBsAg, hepatitis B surface antigen; HCV, hepatitis C virus; HDV, hepatitis D virus; HEV, hepatitis E virus; HIV, human immunodeficiency virus; NAs, nucleoside analogues.





**Figure S2** Calibration curve for the score system


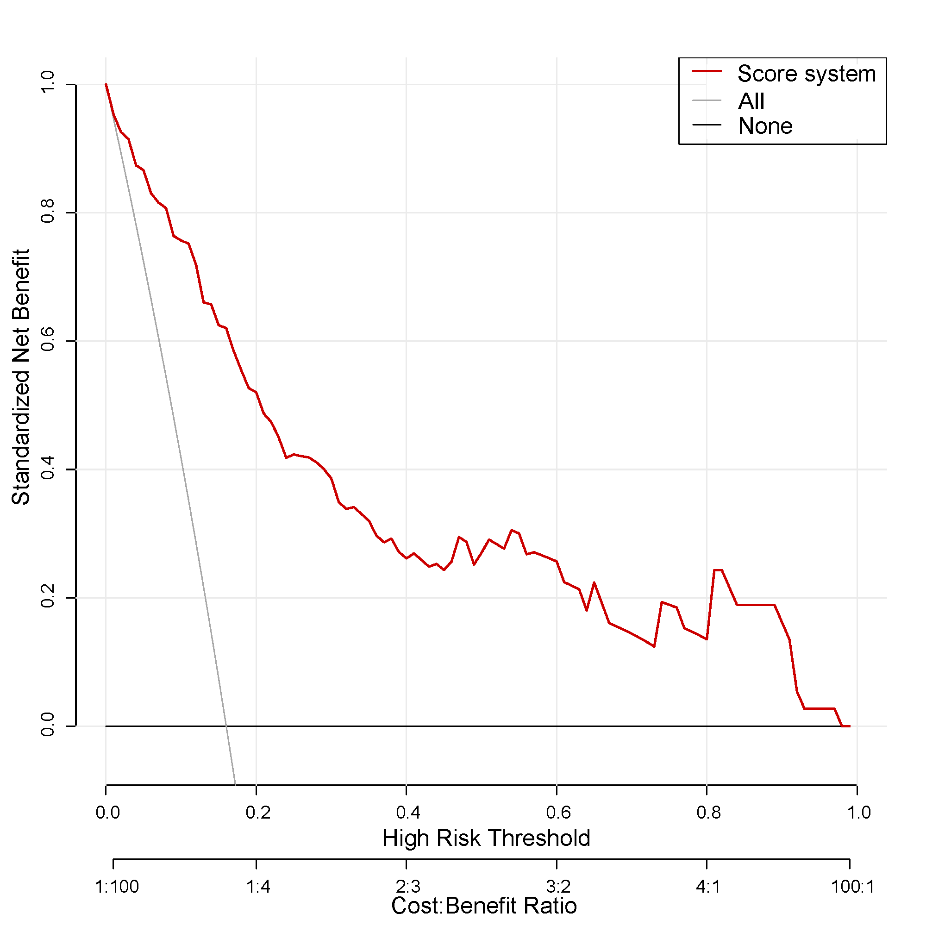


**Figure S3** Decision curve analysis for the score system.

**Table S1** Variables in the predictive score syetem of HBsAg clearance

| predictors | ß-coefficient | Odds ratio(95% CI) | *P* |
| --- | --- | --- | --- |
| Age(years) | -0.070 | 0.933(0.881-0.988) | 0.017 |
| Baseline HBsAg(>605 IU/ml vs ≤605 IU/ml) | 2.332 | 10.296(3.641-29.117) | <0.001 |
| HBsAg decline at week 12(log_10_ IU/ml) | 0.951 | 2.590(1.733-3.869) | <0.001 |
| ALT ratio at week 12(ULN) | 0.374 | 1.453(1.015-2.080) | 0.041 |
| Abbreviations: ALT, alanine aminotransferase; HBsAg, hepatitis B surface antigen; ULN, upper limit of the normal. ALT ratio means ALT/ULN. | | | |
